# Encapsulation and Digestive Evaluation of Infusion Extracts from Semi-Desert Mexican Plants: Phytochemical Profiling and Bioactivities

**DOI:** 10.3390/plants14223448

**Published:** 2025-11-11

**Authors:** Antonio Julián-Flores, Mariela R. Michel, Cristóbal N. Aguilar, Teresinha Gonçalves da Silva, Cristian Torres-León, Juan A. Ascacio-Valdés, Leonardo Sepúlveda, Pedro Aguilar-Zárate, Mónica L. Chávez-González

**Affiliations:** 1 Bioprocesses & Bioproducts Group, Food Research Department, School of Chemistry, Autonomous University of Coahuila, Saltillo 25280, Coahuila, Mexico; antoniojulian@uadec.edu.mx (A.J.-F.); cristobal.aguilar@uadec.edu.mx (C.N.A.); alberto_ascaciovaldes@uadec.edu.mx (J.A.A.-V.); leonardo_sepulveda@uadec.edu.mx (L.S.); 2 Laboratorio Nacional CONAHCYT de Apoyo a la Evaluación de Productos Bióticos (LaNAEPBi), Unidad de Servicio, Tecnológico Nacional de México/I.T. de Ciudad Valles, Ciudad Valles 79010, San Luis Potosí, Mexico; mariela.michel@tecvalles.mx; 3 Department of Antibiotics, Federal University of Pernambuco (UFPE), Recife 54740-520, PE, Brazil; teresinha.goncalves@ufpe.br; 4 Research Center and Ethnobiological Garden, Autonomous University of Coahuila, Viesca 27480, Coahuila, Mexico; ctorresleon@uadec.edu.mx

**Keywords:** medicinal plants, bioactive compounds, antiparasitic activity, encapsulation, simulated digestion

## Abstract

Medicinal plants are widely used in traditional medicine because of their bioactive compounds with therapeutic potential. The semidesert Mexican species *Flourensia cernua*, *Artemisia ludoviciana*, and *Phoradendron californicum* have been traditionally employed as infusions for gastrointestinal disorders. In this study, chemical composition, infusion extraction, phytochemical profiling, antioxidant capacity, and antiparasitic and antibacterial activity were evaluated. The extracts were characterized via HPLC–MS, encapsulated in agar–agar beads, and subjected to in vitro simulated digestion. *A. ludoviciana* presented the highest content of hydrolysable and condensed tannins (5473.33 ± 305.5 mg GAE/100 g; 959.58 ± 14.6 mg CE/100 g, respectively). *F. cernua* presented the highest flavonoid concentration (582.67 ± 16.90 mg CE/100 g). The highest antioxidant activity was observed in *P. californicum* (IC_50_ 74.18 ± 18.43 μg TE/mL in DPPH; IC_50_ 333.38 ± 56.36 μg TE/mL in ABTS). In terms of antiparasitic effects, *A. ludoviciana* extracts presented the lowest IC_50_ value (0.51 ± 0.01 mg/mL), indicating the highest antiparasitic activity. Antibacterial assays revealed that *F. cernua* had the greatest inhibitory effect on *E. coli* (79.60%) and *S. aureus* (78.40%). Encapsulation preserved extract integrity, although simulated digestion resulted in limited compound release, with hydrolysable tannins being the most released. Overall, *P. californicum* presented the strongest antioxidant response, and encapsulation was confirmed as an effective strategy to preserve extract integrity.

## 1. Introduction

In Mexico, the use of medicinal plants has a long-standing tradition and remains the primary option for treating diseases in economically vulnerable populations, particularly in southern states [1,2]. In contrast, the northern semidesert region hosts a wide variety of plants used by rural communities to manage respiratory, cardiovascular, and central nervous system disorders; skin, eye, ear, nose, and oropharyngeal conditions; obstetric, gynecological, and urinary tract diseases; infectious diseases; and gastrointestinal disorders [3,4]. The extreme climatic conditions in which these plants grow induce increased production of phytochemicals, which are associated with biological activities such as antioxidant, anticancer, antidiabetic, and antimicrobial properties. Metabolite production is closely related to the environmental conditions of plant development [5].

*Flourensia cernua* is an endemic species that grows in semiarid regions of México and has traditionally been used to treat digestive disorders. It contains polyphenolic compounds, primarily flavonoids, including chrysin, galangin, apigenin, kaempferol, quercetin derivatives, flavanones, 8-prenyl flavanones, 8-prenyl flavonols, and 5-acetylbenzofurans, as well as sesquiterpenes [6]. *Phoradendron californicum*, commonly known as “toji,” is a hemiparasitic plant that grows on the stems and branches of host plants (mainly *Cercidium* sp. and *Prosopis* sp.), where it photosynthesizes its own carbohydrates. It has been used to treat digestive and stomach disorders, as well as venereal diseases. Some of its identified metabolites include phenolic acids and flavonoids such as gallic acid, catechin, rutin, quercetin, and esculetin [7]. The plant *Artemisia ludoviciana* has been reported to possess antibacterial, antiviral, antiparasitic, antifungal, nematicidal, and insecticidal properties, which contribute to the plant’s defense, in addition to health benefits, due to its high antioxidant content [8]. Despite evidence of bioactive compounds in these plants, conventional extraction methods using nontoxic solvents—comparable to those traditionally applied by indigenous communities—have not yet been explored.

The nutritional and chemical characterization of plants has gained increasing attention, as they are sources of proteins, minerals, vitamins, dietary fiber, carbohydrates, essential fatty acids, phenolic compounds, and secondary metabolites of interest to the food, chemical, and pharmaceutical industries [9]. However, despite their potential, comprehensive studies on their toxicological effects are lacking, and few investigations have explored how bioactive compounds behave within the human body [10]. To preserve the bioactive compounds of plants, different methods are employed, one of which is encapsulation. This process involves protecting the active material (in liquid, solid, or gaseous form) with a coating or wall material, allowing the formation of capsules designed to isolate the core from potential chemical interactions or environmental influences [11]. Encapsulation is among the most widely used strategies in food processing, as it contributes to improving the stability, protection, and bioavailability of bioactive compounds. Products obtained through encapsulation may provide significant health benefits, making them promising ingredients for the development of functional foods [12]. The main objectives of this process are to prevent chemical and physical reactions of the core material and to preserve its biological, functional, and physicochemical properties [13]. The effectiveness of encapsulation can be influenced by several factors, including the methodology applied, the characteristics of the wall and core materials, and the interactions between these compounds [14]. The bioaccessibility of polyphenols is commonly assessed through in vitro gastrointestinal digestion models and is defined as the fraction of the compound released from the food matrix into the gastrointestinal tract that remains available for absorption [15]. The efficiency of polyphenol absorption throughout the gastrointestinal tract is influenced by several factors, including the nature of the food matrix, its solubility, digestibility, the molecular structure of the compounds, and the action of metabolizing enzymes [16]. This type of approach not only enables efficient preliminary analysis of ingredients but also offers considerable advantages, as in vitro techniques are ethically more acceptable, less costly, and less time-consuming than in vivo assays are. Moreover, these systems facilitate the standardization of experimental conditions and the generation of reproducible results [17]. This study aimed to evaluate the chemical composition and bioactive compounds of extracts from *A. ludoviciana*, *F. cernua*, and *P. californicum*, assess their biological activities, and subsequently encapsulate them. The encapsulated extracts were then subjected to a simulated digestion process to determine the release of the compounds.

## 2. Results

### 2.1. Chemical Composition of Studied Plants

The analysis allowed the determination of the chemical composition of the three plants studied. Table 1 shows the macronutrient composition of the plant material, expressed as percentages (%). Carbohydrates were the predominant component in all the species, which is attributed to the plant structure, which is mainly composed of complex carbohydrates such as hemicellulose and cellulose. Compared with the other species analyzed, *P. californicum* presented higher levels of fiber (16.35 ± 0.15%), ash (13.46 ± 0.02%), and protein (5.84 ± 2.60%). On the other hand, *A. ludoviciana* presented a relatively high moisture content of 14.00 ± 0.00%, which may be attributed to the use of whole plants in the analysis, considering that stems contain a relatively high proportion of water. In the case of *F. cernua*, the highest contents of carbohydrates (73.27 ± 0.00%) and fat were observed, with *A. ludoviciana* presenting 6.14 ± 0.59% fat and *F. cernua* presenting 6.99 ± 1.11% fat.

### 2.2. Phytochemical Composition

Phytochemical profiling allowed the quantification of total flavonoids, hydrolysable tannins, and condensed tannins in the three plant species studied. A Tukey test at a 95% confidence interval was performed to compare differences in the phytochemical composition among *Artemisia ludoviciana*, *Flourensia cernua*, and *Phoradendron californicum*.

**Figure 1:**
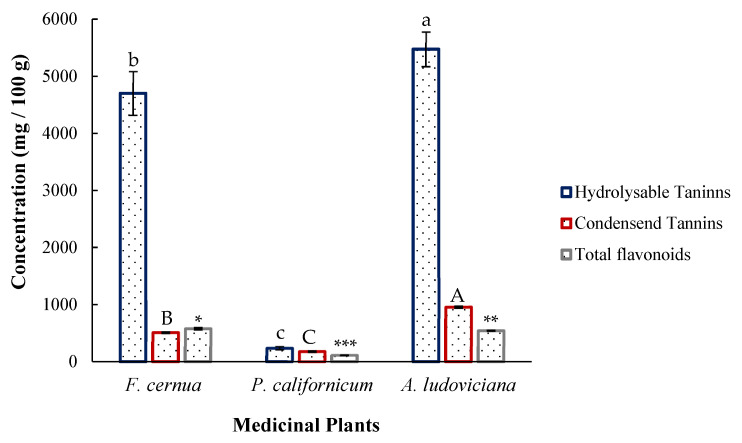
Phytochemical content of medicinal plants. Different letters and asterisks indicate significant differences (Tukey, *p* < 0.05). Lowercase letters correspond to hydrolysable tannins, uppercase letters correspond to condensed tannins, and asterisks correspond to total flavonoids. A smaller number of asterisks indicates a higher concentration.

The results presented in Figure 1 highlight that hydrolysable tannins were the most abundant compounds in all the plants. *Artemisia ludoviciana* presented the highest content, at 5473.33 ± 305.00 mg GAE/100 g. Similarly, the condensed tannin content in *A. ludoviciana* was significantly greater than that in *F. cernua* (513.11 ± 10.00 mg CE/100 g) and *P. californicum* (181.56 ± 9.00 mg CE/100 g), with a value of 959.58 ± 15.00 mg CE/100 g. The total flavonoid content in *F. cernua* was greater, at 582.667 ± 17.00 mg CE/100 g, followed by *A. ludoviciana* at 547.66 ± 5.00 mg CE/100 g and *P. californicum* at 114.333 ± 2.00 mg CE/100 g.

### 2.3. Free Radical–Scavenging Activity

Table 2 shows the antioxidant activity of the plant material, highlighting significant differences among the three species (Tukey, *p* < 0.05). *P. californicum* exhibited the highest activity in both assays, with a median inhibitory concentration (IC_50_) of 74.18 ± 18.43 μg TE/mL and IC_50_ of 333.38 ± 56.36 μg TE/mL for DPPH and ABTS, respectively. Compared with *A. ludoviciana* (IC_50_ 2294.29 ± 307.83 μg TE/mL), *F. cernua* had significantly greater antioxidant activity in the DPPH assay (IC_50_ 1134.56 ± 100.65 μg TE/mL). However, no significant differences (*p* > 0.05) were detected between *F. cernua* (IC_50_ 2926.36 ± 193.85 μg TE/mL) and *A. ludoviciana* (IC_50_ 2973.36 ± 304.29 μg TE/mL) in the ABTS assay.

### 2.4. Antiparasitic Activity

**Figure 2:**
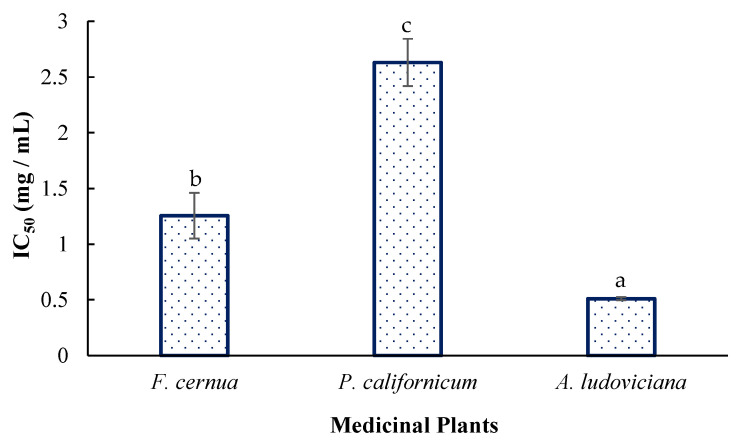
Antiparasitic activity of the plant extracts. Different letters indicate significant differences (Tukey, *p* < 0.05).

According to the comparative statistical analysis (Figure 2), *A. ludoviciana* presented the greatest activity, with an IC_50_ of 0.51 ± 0.01 mg/mL, followed by *F. cernua* (IC_50_ = 1.25 ± 0.20 mg/mL) and *P. californicum* (IC_50_ = 2.63 ± 0.20 mg/mL) (*p* < 0.05).

### 2.5. Inhibition of Bacterial Growth

**Figure 3:**
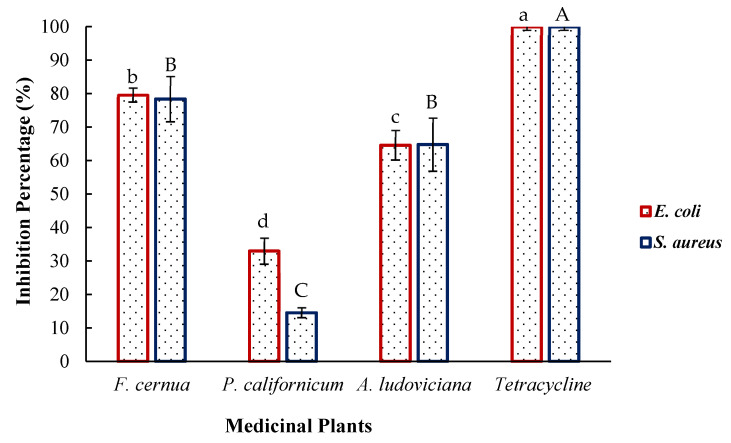
Antibacterial activity of the plant extracts. Different letters indicate significant differences (Tukey, *p* < 0.05). Lowercase letters correspond to *E. coli*, and uppercase letters correspond to *S. aureus*.

The percent inhibition of the pathogenic bacteria *Escherichia coli* and *Staphylococcus aureus* was determined via extracts of the studied medicinal plants. The results are shown in Figure 3, where the antibiotic tetracycline used as a control achieved 100% inhibition at a concentration of 50 μg/mL. *Flourensia cernua* (235.00 ± 19.24 μg GAE/mL) inhibited *E. coli* the most strongly at 79.60 ± 2.00%, followed by *Artemisia ludoviciana* (273.67 ± 15.28 μg GAE/mL) at 64.60 ± 4.00% and *Phoradendron californicum* (120.33 ± 13.66 μg GAE/mL) at 32.90 ± 4.00%. The extracts also inhibited *S. aureus*. However, no significant differences (*p* > 0.05) were detected between *F. cernua* (78.40 ± 7.00%) and *A. ludoviciana* (64.80 ± 8.00%). In contrast, *P. californicum* presented the lowest inhibition (14.60 ± 1.00%).

### 2.6. HPLC–MS Analysis

Table 3 presents the qualitative characterization of the plant material, including its retention time, mass–charge ratio, associated compound, and chemical family. All the compounds are polyphenols, mostly hydroxycinnamic acids, with diverse biological activities.

### 2.7. Agar-Agar Beads

**Figure 4:**
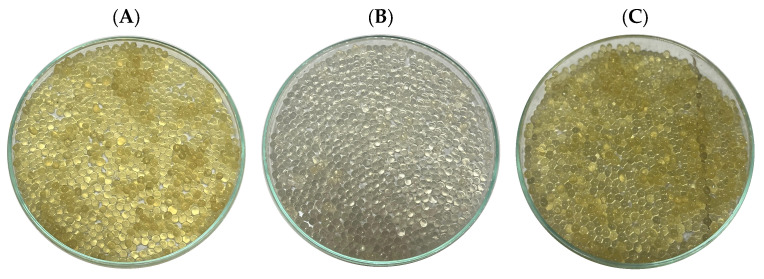
Agar–agar beads: (**A**) *Flourensia cernua*; (**B**) *Phoradendron californicum*; (**C**) *Artemisia ludoviciana*.

This process facilitated the successful encapsulation of infusion extracts from *Artemisia ludoviciana*, *Flourensia cernua*, and *Phoradendron californicum* within agar–agar beads, as illustrated in Figure 4. This approach not only ensures the physical incorporation of the extracts into a stable matrix but also provides a potential strategy for improving their stability, handling, and subsequent controlled release.

### 2.8. In Vitro Digestion of Agar-Agar Encapsulates

**Figure 5:**
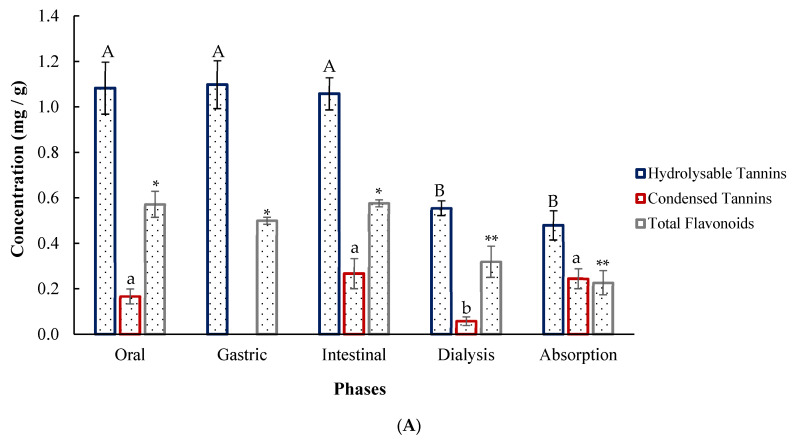

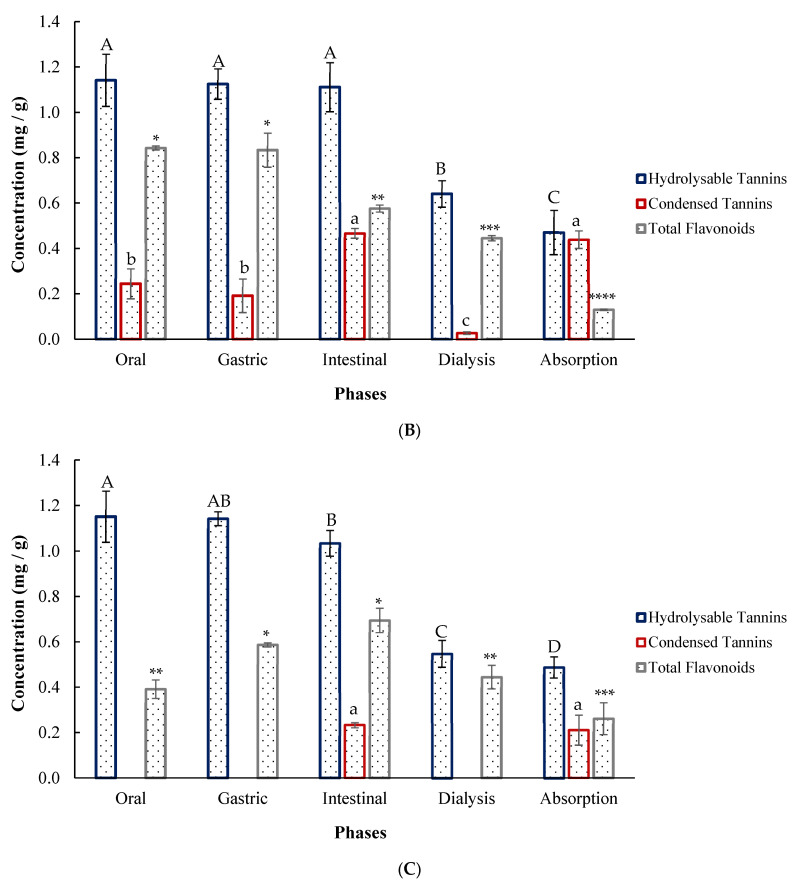
Comparison of encapsulated compound release during in vitro digestion: (**A**) *Flourensia cernua*; (**B**) *Phoradendron californicum*; (**C**) *Artemisia ludoviciana*. Different letters and asterisks indicate significant differences (Tukey, *p* < 0.05). The uppercase letters represent hydrolysable tannins, the lowercase letters represent condensed tannins, and the asterisks represent total flavonoids. A smaller number of asterisks indicates a higher concentration.

Figure 5A shows the phytochemical profile of *F. cernua* during simulated gastrointestinal digestion. Hydrolysable tannins reached the highest concentrations, remaining stable across the oral, gastric, and intestinal phases but decreasing during dialysis, with an estimated absorption of 0.479 ± 0.07 mg/g of encapsulate. Condensed tannins were not detected in the gastric phase, although similar levels were observed in the oral, intestinal, and absorption phases, suggesting potential degradation or interaction with the matrix under acidic conditions. The condensed tannins were almost fully bioaccessible (0.444 ± 0.04 mg/g). The total flavonoid content remained stable in the early phases but decreased during dialysis (0.319 ± 0.07 mg/g), indicating the diffusion of low-molecular-weight compounds across the membrane. In encapsulated *P. californicum* (Figure 5B), hydrolysable tannins showed no significant differences in the first three phases but decreased in the dialysis (0.640 ± 0.05 mg/g) and absorption (0.471 ± 0.09 mg/g) phases. Condensed tannins were highest in the intestinal phase (0.467 ± 0.03 mg/g) and remained bioaccessible in the absorption phase (0.439 ± 0.04 mg/g). Total flavonoids decreased significantly from the oral (0.844 ± 0.01 mg/g) and gastric phases (0.833 ± 0.08 mg/g) to the intestinal phase (0.577 ± 0.01 mg/g), with the lowest value of absorption (0.131 ± 0.00 mg/g) following dialysis (0.446 ± 0.01 mg/g). In encapsulated *A. ludoviciana*, hydrolysable tannins were released in all phases, showing a decreasing trend with a final absorption of 0.487 ± 0.01 mg/g. Condensed tannins were not detected in the oral, gastric, or dialysis phases but were released in the intestinal phase (0.233 ± 0.05 mg/g). The total flavonoid content increased from the oral to the intestinal phase (0.695 ± 0.01 mg/g), reaching the highest concentration, and then decreased during dialysis, indicating potential bioavailability (Figure 5C).

The results of the antioxidant activity are summarized in Table 4. *Phoradendron californicum* exhibited activity in all the digestion phases, as determined by the DPPH assay, with the intestinal phase (60.14 ± 9.66 μg TE/mL) showing the greatest effect, followed by the dialysis phase (55.69 ± 4.19 μg TE/mL). In contrast, for the ABTS assay, activity was detected only in the gastric (75.15 ± 15.38 μg TE/mL) and intestinal phases (238.97 ± 42.89 μg TE/mL). In *Flourensia cernua*, antioxidant activity was quantified by ABTS in the gastric, intestinal, and dialysis phases, with the intestinal phase having the strongest effect (91.46 ± 3.85 μg TE/mL). For *Artemisia ludoviciana*, detectable antioxidant activity was restricted to the gastric phase, with a value of 60.77 ± 15.38 μg TE/mL by ABTS.

## 3. Discussion

### 3.1. Proximal Composition

The results obtained for *F. cernua* are consistent with those reported by Aranda-Ledezma [18], who reported 6.40 ± 2.16% lipids and 67.38 ± 2.88% carbohydrates in the same species. In that study, the protein content was 1.98 ± 0.04%, which was higher than the value obtained in the present work. These differences may be explained by the fact that the plant material was collected in San Jerónimo, Zacatecas, suggesting that geographical origin and environmental conditions influence the macronutrient composition of the species. Scientific evidence exists regarding the nutritional composition of *Artemisia annua*, with reported values of moisture at 11.4%, fiber at 14.2%, and fat at 6.07%, which are comparable to those reported in the present study. In plants of this genus, the composition of these compounds tends to be similar across species [19]. To date, no reports exist on the proximate composition of *P. californicum*. Therefore, this study provides novel information on a plant species that has been scarcely investigated.

### 3.2. Phytochemical Content

*A. ludoviciana* presented a higher content of hydrolysable tannins than González-González [20], with a value of 2560.00 ± 369.00 mg GAE/100 g after 72 h of maceration at room temperature, indicating that greater quantification can be achieved with shorter periods and higher temperatures. The total flavonoid content obtained was approximately four times lower than that reported by Hernández-García [21], who used 2874.50 mg CE/100 g, with ultrasound-assisted extraction using an ethanol/water (70:30 *v*/*v*) mixture for 60 min at 15 min intervals, followed by 24 h of maceration. The combination of cavitation and solvent enhanced flavonoid extraction. For *F. cernua*, the hydrolysable tannin content was lower than that reported by Jasso de Rodríguez et al. [6], who obtained 10,820.00 ± 850.00 mg GAE/100 g via Soxhlet extraction with water as the solvent at a 1:14 solid-to-solvent ratio for 72 h. Although reflux cycles yield greater extraction, the process is much longer than that of a 5 min infusion. With respect to condensed tannins, Alvarez-Pérez et al. [22] reported 0.02 ± 34.00 mg/100 g, indicating high variability and a substantially lower concentration than that obtained in this study, possibly because they used a 2 h maceration (1:10 *w*/*v*) at 60 °C. Although water was also used as the solvent, shorter periods and higher temperatures favor the extraction of the compound. The total flavonoid content obtained for this species was lower than that reported by Aguirre-García et al. [23], who applied a combined ultrasonic–microwave extraction process using 30% ethanol, achieving 12,480.00 ± 690.00 mg QE/100 g. The application of ultrasonic waves and the cavitation process facilitated the release of higher flavonoid concentrations, and subsequent purification allowed for the concentration of these compounds. The values obtained for *P. californicum* were lower than those reported by Assanga et al. [7], who reported 22,500.00 mg GAE/100 g for hydrolysable tannins, 11,800.00 mg CE/100 g for condensed tannins, and 14,800.00 mg/100 g for total flavonoids. These large differences may be due to the infusion process used in their study, which involved a large quantity of plant material (100 g in 1 L), an extraction time of 30 min, and subsequent lyophilization for analysis. Importantly, the entire plant was used, and the extraction time favored the quantification of phenolic compounds.

### 3.3. Antioxidant Activity

*P. californicum* presented greater antioxidant activity than did Méndez-Pfeiffer et al. [24], who reported an IC_50_ of 47.62 ± 2.90 μg/mL in a DPPH assay involving methanol maceration of stems only for three days. The use of methanol as a solvent may have favored the recovery of a greater number of compounds with antioxidant activity. However, the activity did not differ importantly from that obtained with hot water in the present study. Furthermore, in the study by Rodríguez et al. [25], *F. cernua* exhibited an IC_50_ of 69.27 ± 8.90 μg TE/mL in the ABTS assay and 174.37 ± 6.60 μg TE/mL in the DPPH assay after Soxhlet extraction for 72 h. In the case of *A. ludoviciana*, the values obtained indicate lower antioxidant activity than those reported by Younsi et al. [26], who obtained an IC_50_ of 100.00 ± 3.30 μg/mL in the DPPH assay using methanolic extract, and by Wubuli et al. [27] in the ABTS assay, with an IC_50_ of 153.11 ± 8.90 μg/mL. However, these differences are largely attributable to the different species used, namely, *Artemisia herba-alba* and *A. absinthium*. The values reported for *F. cernua* and species of the genus *Artemisia* were considerably greater than those obtained in this study. However, the infusion extraction method employed in the present work allowed for the recovery of antioxidant activity in significantly shorter periods than those previously reported, which describe extraction processes lasting up to three days

### 3.4. Parasiticidal Activity

There is scientific evidence that the essential oils of *A. ludoviciana*, at a concentration of 64 mg/mL, inhibit the proliferation of *Leishmania infantum* promastigotes within the first three hours of incubation, with this activity mainly attributed to camphor, 1,8-cineole, and camphene. In addition, the presence of myricetin was reported and identified in this study via high-performance liquid chromatography (HPLC), which could also contribute to this activity [28]. Furthermore, the efficacy of this plant against adult *Fasciola hepatica* parasites has been demonstrated. The study revealed that at a concentration of 125 mg/L, 90% inhibition of the parasite was achieved after 48 h of incubation. This activity was obtained from an extract prepared by maceration for one week using ethyl acetate as the solvent [29]. The values reported for *F. cernua* and *P. californicum* are highly relevant, as no evidence has been reported to date regarding the antiparasitic activity of these plant species via conventional extraction methods.

### 3.5. Bactericidal Effect

Rodríguez et al. [30] reported 100% inhibition of *E. coli* via ethanol maceration extracts of *Flourensia retinophylla* at 2000 mg/L, demonstrating the antibacterial potential of the genus *Flourensia*. Similarly, scientific evidence indicates that *A. ludoviciana* exhibits antibacterial activity against bacteria such as *H. pylori* and species of the genus *Mycobacterium* [31,32]. Previous studies have reported up to 85% inhibition of *E. coli* using chloroform extracts of *P. californicum*, suggesting that solvent polarity plays a critical role in the recovery of antibacterial metabolites [24]. The antibacterial properties are attributed to the structure of phenolic compounds, particularly their hydroxyl groups. These compounds can interfere with bacterial communication mechanisms by inhibiting quorum sensing, destabilizing or disrupting cell membrane integrity, inhibiting key enzymes in metabolic processes, and generating reactive oxygen species (ROS), which cause microorganisms to lose their intracellular content and disrupt their biological functions [33,34].

### 3.6. Identification of Phytochemicals

The analysis allowed the identification of five compounds in *Flourensia cernua*: caffeic acid, caffeoylquinic acid, isorhamnetin, apigenin, and piceatannol. In the study by Aguirre-García et al. [23], 1-caffeoylquinic acid (*m*/*z* 352.9) and apigenin arabinoside-glucoside (*m*/*z* 562.9) were identified via an ultrasonic–microwave extraction process. In addition, Usme-Duque et al. [35] identified caffeic acid 4-O-glucoside with a mass–charge ratio of 340.8 and a retention time of 5.099 min. These compounds have been reported to exhibit numerous biological activities, including anti-inflammatory, antioxidant, antiaging, antiallergic, antidiabetic, neuroprotective, cardioprotective, and anticancer effects. Additionally, they are effective in the treatment of liver and lung diseases and contribute to maintaining oral and skin health [36,37,38]. In *Phoradendron californicum*, scopoletin was detected, which has been reported for its ability to prevent nausea and vomiting caused by motion sickness, as well as for its antidepressant properties. Additionally, 3- and 4-feruloylquinic acids, which exhibit antioxidant activities and have been shown to protect against oxidative stress and inflammation, have been identified [39,40]. In *Artemisia ludoviciana*, four compounds were found: myricetin, 1-caffeoylquinic acid, 1,3-dicaffeoylquinic acid, and p-coumaroyl tyrosine-9. Kamarauskaite et al. [8] demonstrated the presence of 1-caffeoylquinic acid and 1,3-dicaffeoylquinic acid in purified fractions of acetone extracts obtained through ultrasound-assisted extraction. These compounds are primarily hydroxycinnamic acids and exhibit strong free radical scavenging activity, along with antidiabetic, anti-inflammatory, antimicrobial, antihypercholesterolemic, antihypertensive, and antimutagenic effects. Moreover, myricetin has demonstrated potential anticancer activity by modulating various cellular signaling pathways [41,42].

### 3.7. Simulated Digestion of Encapsulates

As shown in the previous figures, hydrolysable tannins exhibited the greatest release throughout the entire simulated digestive process in all three plant species, followed by total flavonoids and, to a lesser extent, condensed tannins. The released compounds were susceptible to degradation under digestive conditions. Although the degradation of polyphenols in the small intestine has been identified as a key factor limiting their bioaccessibility, the standardized in vitro digestion protocol does not account for the influence of oxygen and bile, despite oxidation being a major mechanism contributing to the deterioration of these compounds [15]. The release profile was consistent with the results obtained from the prior quantification of the extracts before encapsulation, suggesting that the release of phenolic compounds during in vitro digestion is proportional to their initial concentration. Notably, the protocol does not include a mastication phase, which could affect the disintegration of the beads and increase the liberation of phenolic compounds from the encapsulating matrix. The encapsulation matrix itself plays a crucial role in phytochemical release, as the chemical structure of agar–agar—composed of agaropectin and agarose—may establish hydrogen bonding and hydrophobic interactions with polyphenols, thereby hindering their diffusion into the digestive medium [43]. On the other hand, although *P. californicum* demonstrated measurable antioxidant activity across multiple phases, the absence of activity in some phases of *A. ludoviciana* and *F. cernua* suggests that the low concentration of phenolic compounds released during digestion was insufficient to exert a detectable effect. These results indicate that hydrolysable tannins and total flavonoids are the main compounds responsible for the observed antioxidant effects. The activity can be attributed to specific compounds identified in the infusion extracts, including 1-caffeoylquinic acid, apigenin arabinoside-glucoside, 3-feruloylquinic acid, and myricetin. This effect may be related to their higher solubility and bioavailability, whereas the limited release of condensed tannins could reduce their contribution to the overall bioactivity of the encapsulated extracts. Notably, the antioxidant activity of these species has not been previously reported, highlighting the importance of this study in providing novel scientific evidence.

## 4. Materials and Methods

### 4.1. Plant Material Pretreatment and Extraction

The plants *Flourensia cernua* and *Phoradendron californicum* were collected in Parras, Coahuila, México (25°33′51.5″ N 102°33′54.5″ W), from November to December 2023. *Artemisia ludoviciana* was purchased from a local herbal market (Centro Botánico Azteca, Saltillo, Coahuila, México) in December 2023. The plants were identified by Dr. Cristian Torres León from the Research Center and Ethnobiological Garden (CIJE). For *F. cernua* and *P. californicum*, only the leaves were used, whereas for *A. ludoviciana,* the whole plant was used. The raw material was dried at 60 °C for 24 h (NAPCO 1000 incubator, National Appliance Co., Portland, OR, USA). For the preparation of the extracts, 100 mL of distilled water was added to a beaker and heated to 100 °C. Subsequently, 5 g of each plant material was infused for 5 min. The mixtures were then filtered by gravimetry using a funnel and Whatman No. 42 filter paper (Cytiva^®^, Marlborough, MA, USA).

### 4.2. Proximal Composition of Medicinal Plants

The proximal analysis of the raw material was carried out under the guidelines of AOAC International [44]. The moisture, fat (AOAC 920.39C), total crude fiber (AOAC 962.09), total protein (AOAC 954.01), ash, and carbohydrate contents were determined. The moisture content of each sample was determined by drying to a constant weight in an oven at 100 °C to measure water loss. Fat extraction was carried out via the Soxhlet method, with hexane used as the solvent. The crude fiber content was measured via acid and alkaline digestion methods. The protein content was quantified via the Kjeldahl method, with a nitrogen-to-protein conversion factor of 6.25. The ash content was determined by the weight difference after incineration in a muffle furnace at 600 °C. Carbohydrates were measured as percentages of the other components.

### 4.3. Phytochemicals Quantification

#### 4.3.1. Hydrolysable Tannins

The determination of hydrolysable tannins via the Folin–Ciocalteu method involved preparing a stock solution of gallic acid for the calibration curve, which ranged from 200 ppm. A total of 20 µL of each sample was diluted 1:10 (100 µL of sample and 900 µL of water), 20 µL of the Folin–Ciocalteu reagent was added, and the mixture was incubated for 5 min at room temperature. After this time, 20 µL of 0.01 M sodium carbonate was added, the sample was incubated for 5 min at room temperature, and finally, 125 µL of water was added; the samples were deposited in a microplate for absorbance reading at 734 nm [45].

#### 4.3.2. Condensed Tannins

For the determination of condensed tannins via the HCl-butanol method, a catechin stock solution was prepared for determination of the calibration curve up to 1000 ppm (in triplicate). Five hundred microliters of each sample was diluted 1:10 (100 µL of sample and 900 µL of water), 3 mL of HCl-butanol (1:9) was added, 0.1 mL of ferric reagent was added (20 mL of concentrated HCl and 2 drops of solid aluminum ferric sulfate to reach 100 mL), and the tubes were placed in a water bath at 100 °C for one hour. Finally, each sample was placed in a microplate, and the absorbance was read at 460 nm [45].

#### 4.3.3. Total Flavonoids

The determination of total flavonoids was carried out according to the methodology of De la Rosa et al. [46] with minor modifications. A total of 310 µL of extract was mixed with 93 µL of 5% NaNO_3_ and 93 µL of distilled water. The mixture was vortexed and incubated for 5 min. Subsequently, 93 µL of 10% AlCl_3_ was added, and the mixture was incubated for 3 min. Finally, 125 µL of NaOH (0.5 M) was added, and the mixture was incubated for 30 min at room temperature in the absence of light. The absorbance was measured at 510 nm via a spectrophotometer (BIOBASE-EL10A, Biobase Biodustry, Jinan, China). The total flavonoid content was calculated via a catechin calibration curve (1000 to 100 ppm). All the assays were performed in triplicate.

### 4.4. Antioxidant Activity

#### 4.4.1. DPPH Assay

The free radical reagent 2,2-diphenyl-1-picrylhydrazyl (DPPH) was prepared at a concentration of 60 µM in methanol. Subsequently, 7 µL of the sample (extracts) was mixed with 193 µL of the DPPH solution in methanol in a 96-well microplate at room temperature and left to stand for 30 min. Absorbance was measured at 517 nm via a microplate reader (BIOBASE-EL10A, China). The results were calculated and expressed as Trolox equivalents per milliliter. The DPPH reagent in methanol was used as a control [47].

#### 4.4.2. ABTS Assay

The evaluation of ABTS•+ radical inhibition was conducted according to the methodology proposed by Aranda-Ledezma et al. [48], with slight modifications. The ABTS•+ radical cation was generated by mixing an aqueous solution of ABTS•+ (7 mmol·L^−1^) with potassium persulfate (2.45 mmol·L^−1^) in the dark at room temperature for 12 h prior to use. The working solution of ABTS•+ was prepared in ethanol with an absorbance of 0.700 ± 0.002 nm at 734 nm. A volume of 5 µL of the sample was mixed with 95 µL of the ABTS•+ solution to initiate the reaction. After 1 min of reaction, the absorbance was measured at 734 nm. The results are expressed as Trolox equivalents (TE µg·mL^−1^) on the basis of a calibration curve prepared with the same standard.

### 4.5. Antileishmanial Activity

The antiparasitic activity of the extracts was evaluated via the promastigote form of *Leishmania braziliensis* LTB (MHOM/BR/75/M2903) (10^6^ cells/mL) according to the methodology described by Pereira et al. [49], with minor modifications. Briefly, 50 μL of Schneider’s medium was added to each well of a 96-well microplate, followed by 50 μL of the sample, which was subsequently serially diluted (starting from 50 mg/mL). Then, 50 μL of parasite suspension was added, and the plates were incubated at 26 °C for 24 h. Cell growth was determined by counting in a Neubauer chamber (Brand GmbH, Berlin, Germany). The concentration required to inhibit promastigote growth by 50% (IC_50_) was estimated through linear regression analysis. Each assay was performed in quintuplicate across three independent experiments.

### 4.6. Antibacterial Activity

The antibacterial potential of the plant material was assessed by determining the inhibition of *S. aureus* and *E. coli*, as described by Haq et al. [50]. In brief, 100 µL of Mueller–Hinton broth was added to each well of the 96-well microdilution tray, with the exception of the sterility control. Then, 100 µL of the extract was added to each well, except for those that served as negative and drug-free controls. Then, 10 µL of bacterial suspension (approximately 2–8 × 10^5^ CFU/mL) was poured into each well of the microtiter plates, except for the negative and sterile control wells. Tetracycline was used as the standard antibiotic against *Escherichia coli* (ATCC 25922) and *Staphylococcus aureus* (ATCC 25923) at an initial concentration of 128 µg/mL and was serially diluted to 0.5 µg/mL according to the Clinical and Laboratory Standards Institute 2021 recommendations. The microtiter plates were incubated for 18–24 h at 37 °C. The minimum inhibitory concentration (MIC) was determined as the lowest concentration of the antimicrobial agent that inhibited visible bacterial growth in the wells. To determine the MICs, the turbidity of the plates was visually examined and compared with that of the control well.

#### Spectrophotometric Broth Microdilution Method

In addition to visual interpretation, the spectrophotometric broth microdilution method was also used to analyze bacterial growth inhibition. After the microtiter plate was loaded, the optical density (OD) was measured via a microplate reader (BIOBASE-EL10A, China) at 620 nm. The OD of each well was taken before incubation (t0) and after 24 h of incubation (t24). Any negative value was assigned a value of zero, whereas values greater than 100 were taken as accurate values of 100. The following formula was used for analysis of growth inhibition:

Grown Inhibition (%) = 100 − ((OD test t24 − t0/OD control t24 − t0) × 100)(1)

### 4.7. HPLC-MS Analysis

The plant extracts were characterized by reversed-phase liquid chromatography according to the methodology described by Ascacio-Valdés et al. [51]. The system consisted of an autosampler (Varian ProStar 410, Palo Alto, CA, USA), a ternary pump (Varian ProStar 230I, Palo Alto, CA, USA), and a PDA detector (Varian ProStar 330, Atlanta, CA, USA). A liquid chromatography ion trap mass spectrometer (Varian 500-MS IT mass spectrometer, Palo Alto, CA, USA) was also utilized with an electrospray ion source. The samples (5 µL) were injected onto a Denali C18 column (150 × 2.1 mm, 3 µm, Grace, Palo Alto, CA, USA). The oven temperature was maintained at 30 °C. The eluents were formic acid (0.2%, *v*/*v*; solvent A) and acetonitrile (solvent B). The following gradient was applied: initial, 3% B; 0–5 min, 9% B linear; 5–15 min, 16% B linear; and 15–45 min, 50% B linear. The column was then washed and reconditioned. The flow rate was maintained at 0.2 mL min^−1^, and elution was monitored at 254, 280, 320, and 550 nm. The whole effluent (0.2 mL min^−1^) was injected into the source of the mass spectrometer without splitting. All MS experiments were carried out in negative mode [M−H]^−^. Nitrogen was used as the nebulizing gas, and helium was used as the damping gas. The ion source parameters were as follows: the spray voltage was 5.0 kV, and the capillary voltage and temperature were 90.0 V and 350 °C, respectively. The data were collected and processed via MS Workstation software (Version 6.9). The samples were first analyzed in full-scan mode in the *m*/*z* range of 50–2000.

### 4.8. Encapsulation

The extracts were encapsulated in agar beads (Alday Ingredientes SA de CV, San Pedro Cholula, Puebla, México) via a BÜCHI B-390 Encapsulator (BÜCHI Labortechnik AG, Flawil, Switzerland) following the manufacturer’s protocol (Application Note No. 137/2013) developed in-house by BÜCHI Labortechnik AG, with minor modifications (review the Supplementary Material Section). Briefly, 4 g of agar powder was dissolved in distilled water (100 mL) at 65 °C, continuously stirred, and maintained in a water bath to avoid solidification. The plant extracts (100 mL) were incorporated into the agar solution, which was pumped through a heated nozzle (70 °C) of the encapsulator and broken into droplets by an ultrasonic vibration frequency. The droplets were collected in a jacket reactor containing medium-chain triglyceride (canola oil) maintained at 10 °C with a recirculating chiller (BÜCHI F-305, BÜCHI Labortechnik AG, Flawil, Switzerland) and vigorously stirred to ensure bead hardening before reaching the bottom of the vessel. The beads were hardened for 30 min in refrigerated oil (<10 °C), filtered, washed with distilled water, and dried via a rotary evaporator (BÜCHI R-100, BÜCHI Labortechnik AG, Flawil, Switzerland) under constant rotation to prevent agglomeration.

### 4.9. In Vitro Simulated Gastrointestinal Digestion

In vitro digestion was simulated following the methodology of Gómez-García et al. [17] with modifications. The procedure involved pH variation, temperature control, peristaltic movements, enzymatic action (utilizing pancreatic enzymes and bile salts), and dialysis to simulate intestinal absorption. The enzymatic solutions were freshly prepared under refrigeration prior to use. Digestion was carried out in a shaker (Innova 44, Eppendorf/New Brunswick Scientific, Hamburg, Germany) at 37 °C with constant agitation to simulate physiological conditions. Aliquots (1 mL) were collected at the end of each phase, frozen, and later analyzed for phenolic compounds and antioxidant capacity before and after simulated digestion.

#### 4.9.1. Simulated Oral Digestion

The initial pH of each sample was adjusted to 5.6–6.9 using 0.1 M NaOH. Oral digestion was performed via the addition of 0.3 mL of α-amylase solution (100 U/mL in 1 mM CaCl_2_, Sigma-Aldrich, St. Louis, MO, USA), and the mixture was incubated for 2 min at 200 rpm.

#### 4.9.2. Simulated Gastric Digestion

For gastric digestion, the pH of the samples was adjusted to 2.0 using 1 M HCl. A pepsin solution (25 mg/mL in 0.1 N HCl; Sigma-Aldrich, St. Louis, USA) was added at a ratio of 0.05 mL per mL of sample to simulate gastric juice. The mixture was incubated for 1 h under agitation at 130 rpm.

#### 4.9.3. Simulated Intestinal Digestion

Small intestine digestion was performed by adjusting the pH to 6.0 with 1 M NaHCO_3_. The intestinal juice was simulated by dissolving 1.33 g of Espavén^®^ enzymatic preparation (Bausch Health Companies Inc., Laval, QC, Canada) in 100 mL of 0.1 M NaHCO_3_. The mixture was added at a ratio of 0.25 mL per mL of sample, and the solution was incubated for 2 h at 45 rpm.

#### 4.9.4. Small Intestine Absorption—Dialysis

To simulate phenolic compound absorption, dialysis membranes (6–8 kDa) were prepared by cutting segments with an extra 10–20% length to allow flotation. The membranes were hydrated in distilled water at room temperature for 30 min to remove preservatives and then rinsed with distilled water. For the dialysis setup, a beaker was filled with 500 mL of dialysate solution (50 mM phosphate buffer, pH 7–7.5) at a sample-to-dialysate ratio of 1:100. The rinsed dialysis membrane was sealed at one end with plastic clamps 3–5 cm from the bottom, and 5 mL of the digested small intestine sample was added through the open end. The top was secured at least 5 cm from the end to allow flotation. The membrane was placed in phosphate solution and maintained at 37 °C with agitation at 150 rpm. After 24 h, aliquots of the “IN” fraction (inside the dialysis membrane) and “OUT” fraction (outside the membrane) were collected to quantify the phytochemical content and antioxidant activity. The “IN” fraction represents nonabsorbable compounds potentially reaching the colon, whereas the “OUT” fraction represents potentially absorbable compounds. The samples were kept in darkness and frozen until analysis.

## 5. Conclusions

The studied plants, *Artemisia ludoviciana*, *Flourensia cernua*, and *Phoradendron californicum,* contain phytochemical compounds such as hydrolysable tannins, total flavonoids, and condensed tannins. Analysis of the proximal chemical composition revealed a high carbohydrate content in all the evaluated species. The infusion extracts exhibited important antioxidant, antimicrobial, and antiparasitic activities. Qualitative characterization by HPLC–MS identified compounds of interest that are associated with the observed biological activities. The encapsulation process successfully produced agar beads containing the infusion extracts of the plants studied. Furthermore, the in vitro simulated digestion of the beads resulted in limited release of phytochemical compounds; however, they still exhibited antioxidant activity.

This study provides evidence supporting the botanical knowledge of indigenous communities, demonstrating that traditional methods of plant use can effectively extract phytochemical compounds with significant biological activities, including antioxidant, antibacterial, and antiparasitic effects. These findings highlight the scientific importance of ethnobotanical practices and their potential application in the development of bioactive natural products via conservation technologies such as encapsulation.

## Figures and Tables

**Table 1:** Proximal composition (%) of medicinal plants: component-wise comparison (Tukey, *p* < 0.05).

Component (%)	*A. ludoviciana*	*F. cernua*	*P. californicum*
Moisture	14.00 ± 0.00 ^a^	0.80 ± 0.00 ^b^	0.40 ± 0.00 ^b^
Fat	6.14 ± 0.59 ^a^	6.99 ± 1.11 ^a^	3.22 ± 0.10 ^b^
Fiber	13.65 ± 0.20 ^b^	8.58 ± 0.20 ^c^	16.35 ± 0.15 ^a^
Protein	3.65 ± 0.33 ^ab^	1.17 ± 1.08 ^b^	5.84 ± 2.60 ^a^
Ash	9.7 ± 0.00 ^b^	9.19 ± 0.01 ^b^	13.46 ± 0.02 ^a^
Carbohydrates	52.86 ± 0.00 ^c^	73.27 ± 0.00 ^a^	60.73 ± 0.00 ^b^

Means comparison test was conducted by comparing within components. Letters indicate statistical groupings, allowing rapid identification of means that differ significantly or are statistically similar.

**Table 2:** Antioxidant activity of the plant extracts expressed as IC_50_.

Assay	*A. ludoviciana*	*F. cernua*	*P. californicum*
DPPH	2294.29 ± 307.83 ^c^	1134.56 ± 100.65 ^b^	74.18 ± 18.43 ^a^
ABTS	2973.36 ± 304.29 ^b^	2926.36 ± 193.85 ^b^	333.38 ± 56.36 ^a^

Means comparison test were conducted among the plant species. Different letters indicate significant differences (Tukey, *p* < 0.05).

**Table 3:** Chemical characterization of the compounds in medicinal plant extracts via HPLC–MS.

Plant	Retention Time (min)	Mass [M−H]^−^	Compound	Family
*Artemisia ludoviciana*	5.238	317	Myricetin	Flavonols
	7.582	352.9	1-Caffeoylquinic acid	Hydroxycinnamic acids
	32.039	515	1,3-Dicaffeoylquinic acid	Hydroxycinnamic acids
	44.778	327.1	p-Coumaroyl tyrosine	Hydroxycinnamic acids
*Flourensia cernua*	5.289	341	Caffeic acid 4-O-glucoside	Hydroxycinnamic acids
	19.413	353	1-Caffeoylquinic acid	Hydroxycinnamic acids
	21.569	623	Isorhamnetin 3-O-glucoside 7-O-rhamnoside	Methoxyflavonols
	24.263	563	Apigenin arabinoside-glucoside	Flavones
	43.875	243.1	Piceatannol	Stilbenes
*Phoradendron californicum*	6.255	190.9	Scopoletin	Hydroxycoumarins
	18.99	366.9	3-Feruloylquinic acid	Methoxycinnamic acids
	24.31	366.9	4-Feruloylquinic acid	Methoxycinnamic acids

**Table 4:** Antioxidant activity during simulated digestion of the encapsulated extracts (Tukey, *p* > 0.05).

Plant	Phases	DPPH (μg TE/mL)	ABTS (μg TE/mL)
*Artemisia* *ludoviciana*	Oral	ND	ND
	Gastric	ND	60.77 ± 15.38 ^a^
	Intestinal	ND	ND
	Dialysis	ND	ND
*Flourensia* *cernua*	Oral	ND	ND
	Gastric	ND	99.23 ± 15.38 ^ab^
	Intestinal	ND	91.46 ± 3.85 ^a^
	Dialysis	ND	114.62 ± 0.8 ^b^
*Phoradendron* *californicum*	Oral	81.53 ± 4.59 ^c^	ND
	Gastric	109.03 ± 3.76 ^b^	75.15 ± 15.38 ^a^
	Intestinal	60.14 ± 9.66 ^a^	238.97 ± 42.89 ^b^
	Dialysis	55.69 ± 4.19 ^a^	ND

ND: Not detected. Letters indicate statistical groupings, providing a quick visual reference for significant differences among means.

## Data Availability

The raw data supporting this study are included in the article. Further inquiries can be directed to the corresponding authors.

## Supplementary Materials

The following supporting information can be downloaded at: https://www.mdpi.com/article/10.3390/plants14223448/s1, Figure S1. Encapsulation process.

## Abbreviations

The following abbreviations are used in this manuscript:

IC_50_	Media Inhibitory Concentration
CFU	Colony-Forming Unit
MIC	Minimum Inhibitory Concentration
OD	Optical Density
HPLC-MS	High-Performance Liquid Chromatography–Mass Spectrometry
