# Supplementary material for: Encapsulation and Digestive Evaluation of Infusion Extracts from Semi-Desert Mexican Plants: Phytochemical Profiling and Bioactivities
Source: Plants (Basel). 2025 Nov 11;14(22):3448. doi: 10.3390/plants14223448 (PMC12655835; doi:10.3390/plants14223448)
Supplement: Supplementary file 1 [file plants-14-03448-s001.zip › plants-3931498-supplementary.pdf]

plants

MDPI

Article

Encapsulation and Digestive Evaluation of Infusion Extracts from Semi-Desert Mexican Plants: Phytochemical Profiling and Bioactivities

Antonio Julián-Flores [1], Mariela R. Michel [2], Cristóbal N. Aguilar [1], Teresinha Gonçalves da Silva [3], Cristian Torres-León [4], Juan A. Ascacio-Valdés [1], Leonardo Sepúlveda [1], Pedro Aguilar-Zárate [2,*] and Mónica L. Chávez-González [1,*]

[1] Bioprocesses & Bioproducts Group, Food Research Department, School of Chemistry, Autonomous University of Coahuila, Saltillo 25280, Coahuila, Mexico; antoniojulian@uadec.edu.mx (A.J.-F.); cristobal.aguilar@uadec.edu.mx (C.N.A.); alberto_ascaciovaldes@uadec.edu.mx (J.A.A.-V.); leonardo_sepulveda@uadec.edu.mx (L.S.)
[2] Laboratorio Nacional CONAHCYT de Apoyo a la Evaluación de Productos Bióticos (LaNAEPBi), Unidad de Servicio, Tecnológico Nacional de México/I.T. de Ciudad Valles, Ciudad Valles 79010, San Luis Potosí, Mexico; mariela.michel@tecvalles.mx
[3] Department of Antibiotics, Federal University of Pernambuco (UFPE), Recife 54740-520, PE, Brazil; teresinha.goncalves@ufpe.br
[4] Research Center and Ethnobiological Garden, Autonomous University of Coahuila, Viesca 27480, Coahuila, Mexico; ctorresleon@uadec.edu.mx
* Correspondence: pedro.aguilar@tecvalles.mx (P.A.-Z.); monica_chavez@uadec.edu.mx (M.L.C.-G.)

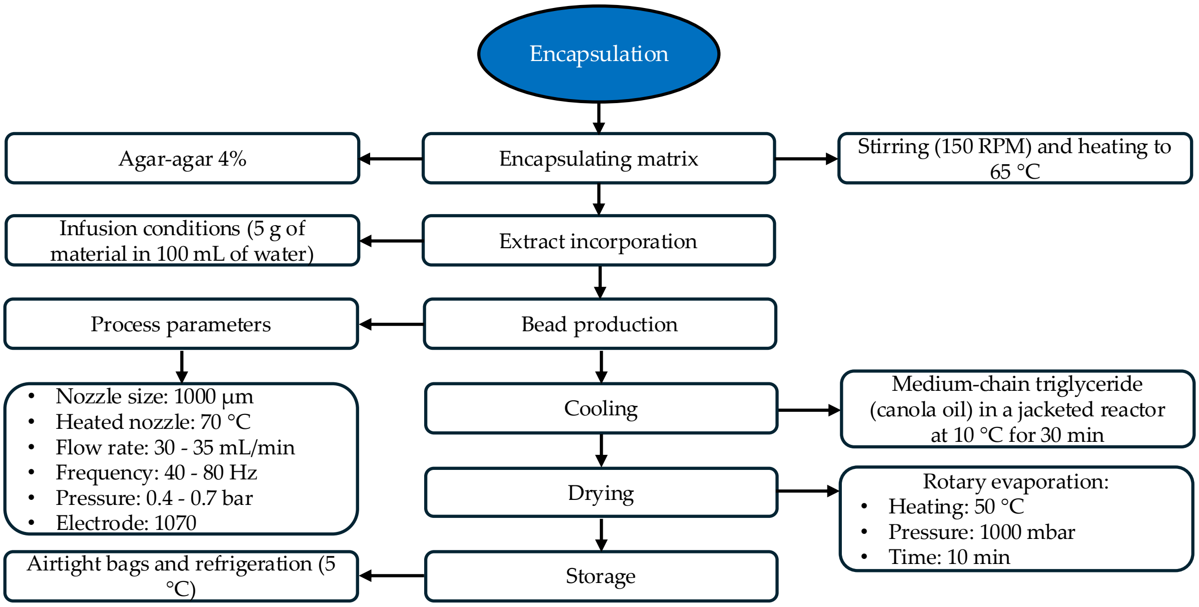

Figure S1. Encapsulation process.
